# Anthropometric measures and HbA1c to detect dysglycemia in young Asian women planning conception: The S-PRESTO cohort

**DOI:** 10.1038/s41598-020-66147-x

**Published:** 2020-06-08

**Authors:** Anne H. Y. Chu, Izzuddin M. Aris, Sharon Ng, See Ling Loy, Jonathan Y Bernard, Mya Thway Tint, Wen Lun Yuan, Keith M. Godfrey, Jerry Kok Yen Chan, Lynette Pei-Chi Shek, Yap Seng Chong, Kok Hian Tan, Seng Bin Ang, Heng Hao Tan, Bernard S. M. Chern, Fabian Yap, Yung Seng Lee, Ngee Lek, Melvin Khee-Shing Leow, Chin Meng Khoo, Shiao-Yng Chan

**Affiliations:** 1 Singapore Institute for Clinical Sciences (SICS), Agency for Science, Technology and Research (A*STAR), Singapore, Singapore; 2 Department of Obstetrics and Gynaecology, Yong Loo Lin School of Medicine, National University of Singapore, Singapore, Singapore; 3 Division of Chronic Disease Research Across the Lifecourse, Department of Population Medicine, Harvard Medical School and Harvard Pilgrim Health Care Institute, Boston, Massachusetts USA; 4 Department of Reproductive Medicine, KK Women’s and Children Hospital, Singapore, Singapore; 5 Duke-NUS Medical School, Singapore, Singapore; 6 Centre for Research in Epidemiology and Statistics, Inserm, Villejuif, France; 7 Department of Paediatrics, Yong Loo Lin School of Medicine, National University of Singapore, Singapore, Singapore; 8 MRC Lifecourse Epidemiology Unit and NIHR Southampton Biomedical Research Centre, University of Southampton and University Hospital Southampton NHS Foundation Trust, Southampton, UK; 9 Department of Maternal Fetal Medicine, KK Women’s and Children’s Hospital, Singapore, Singapore; 10 Department of Family Medicine Service, KK Women’s and Children’s Hospital, Singapore, Singapore; 11 Division of Obstetrics & Gynaecology, KK Women’s and Children’s Hospital, Singapore, Singapore; 12 Lee Kong Chian School of Medicine, Nanyang Technological University, Singapore, Singapore; 13 Department of Paediatrics, KK Women’s and Children’s Hospital, Singapore, Singapore; 14 Department of Medicine, Yong Loo Lin School of Medicine, National University of Singapore, Singapore, Singapore

**Keywords:** Epidemiology, Diagnostic markers, Endocrine system and metabolic diseases

## Abstract

We investigated whether adding anthropometric measures to HbA1c would have stronger discriminative ability over HbA1c alone in detecting dysglycemia (diabetes and prediabetes) among Asian women trying to conceive. Among 971 Singaporean women, multiple regression models and area under receiver-operating characteristic (AUROC) curves were used to analyze associations of anthropometric (weight, height, waist/hip circumferences, 4-site skinfold thicknesses) and HbA1c z-scores with dysglycemia (fasting glucose ≥6.1 mmol/L with 2-hour glucose ≥7.8 mmol/l). The prevalence of dysglycemia was 10.9%. After adjusting for sociodemographic/medical history, BMI (Odds Ratio [OR] = 1.62 [95%CI 1.32–1.99]), waist-to-height ratio (OR = 1.74 [1.39–2.17]) and total skinfolds (OR = 2.02 [1.60–2.55]) showed the strongest associations with dysglycemia but none outperformed HbA1c (OR = 4.09 [2.81–5.94]). After adjustment for history, adding BMI, waist-to-height ratio and total skinfolds (anthropometry trio) as continuous variables to HbA1c (AUROC = 0.80 [95%CI 0.75–0.85]) performed similarly to HbA1c alone (AUROC = 0.79 [0.74–0.84]). However, using clinically-defined thresholds without considering history, as in common clinical practice, BMI ≥ 23 kg/m^2^ + HbA1c ≥ 5.7% (AUROC = 0.70 [0.64–0.75]) and anthropometry trio + HbA1c ≥ 5.7% (AUROC = 0.71 [0.65–0.76]) both outperformed HbA1c ≥ 5.7% alone (AUROC = 0.61 [0.57–0.65]). In a two-stage strategy, incorporating BMI ≥ 23 kg/m^2^ alongside HbA1c ≥ 5.7% into first-stage screening to identify high risk women for subsequent oral glucose tolerance testing improves dysglycemia detection in Asian women preconception.

## Introduction

The prevalence of diabetes has increased dramatically with the global age-standardized prevalence nearly doubling from 4.7% in 1980 to 8.5% in 2014, with similar estimates within South-East Asia^1^. Of concern is that in Singapore, alongside other newly industrialized nations such as South Korea and Hong Kong, a sizeable proportion of 31.9–83.8% of people with diabetes remain undiagnosed^2^. The increasing prevalence is also seen among reproductive age women^3^, which leads to a higher risk of complications from longer life exposure to hyperglycemia.

It was suggested that adult diabetes stemmed from their early life exposure to a hyperglycemic environment *in utero*^4^. When pregnancies are complicated by hyperglycemia, this can predispose offspring to future diabetes, obesity and cardiovascular diseases, thereby generating the intergenerational cycle of diabetes-begetting-diabetes^5^. Improving preconception health is therefore crucial in preventing diabetes in offspring and represents a critical window of opportunity for intervention. The St Vincent Declaration (1989) set a goal to achieve similar pregnancy outcomes in women with diabetes to those without^6^, with a key point being detection of diabetes and optimization of glycemic control preconception. Similarly there is mounting evidence that even milder degrees of preconception dysglycemia (including pre-diabetes) may also predispose the offspring to adverse long-term health consequences^7^. However, approximately half of all pregnancies are unplanned and approximately 20% of births worldwide are unintended^8^; and if planned, only a minority of women seek preconception care. Even then, standard preconception care does not include routine screening for dysglycemia (diabetes and prediabetes).

Screening for preconception dysglycemia is opportunistic, subject to the background population risk and physician bias. The oral glucose tolerance test (OGTT) is the current gold standard in the diagnosis of dysglycemia^9^, but the adoption of universal screening with OGTT preconception is challenging due to staffing costs, patient inconvenience, increased patient time in clinics, the dislike of overnight fasting, taste of the glucose drink and unproven cost-effectiveness. There is thus a need to search for a more acceptable alternative approach to first line screening for preconception dysglycemia such that only women identified as high risk are offered a diagnostic OGTT.

Glycated hemoglobin (HbA1c) is a relatively simple test requiring only a single non-fasting blood sample. HbA1c is a strong marker for future diabetes, but its sensitivity in detecting concurrent dysglycemia is relatively poor in some populations^10^. The sensitivity and specificity of HbA1c are highly affected by ethnicity, age and hemoglobinopathies^11^, which are prevalent among Asians^12^. Furthermore, the use of HbA1c threshold of ≥5.7% as an indicator of individuals at risk of diabetes and pre-diabetes was determined from general populations of predominantly the middle-aged, diverse ethnicities, and both sexes^13,14^, and is currently not adopted widely as a screening test in the preconception setting. HbA1c, even if performed, is often not interpreted alongside sociodemographic and clinical history (hereafter collectively referred to as history) in determining those who should subsequently undergo an OGTT for definitive diagnosis of dysglycemia.

Obesity, a major risk factor for dysglycemia, is often assessed by BMI; whilst other anthropometric parameters (e.g. waist circumference [WC], waist–hip ratio [WHR], waist-height ratio [WHtR]) are less commonly utilized in clinical practice. There is evidence suggesting that variation in the magnitude of association between obesity and diabetes depends on the type of anthropometry used to measure obesity^15^. Abdominal fat, estimated with WC and WHR, is associated with increased diabetes risk. WC is shown to be a better predictor of various indices of glucose-insulin homeostasis than BMI in Western populations^16^, whereas WHR is weakly associated with glucose tolerance^17^. A Body Shape Index (ABSI), calculated using WC, height, weight, demonstrated superiority over BMI in predicting diabetes in US populations^18^. Lastly, skinfold-thicknesses (truncal, peripheral) as complementary adiposity measures have also been explored for predicting abnormal glucose tolerance^19^.

We performed a cross-sectional analysis of baseline preconception data from a prospective cohort to examine the discriminative ability of various anthropometric measures and HbA1c to detect dysglycemia in Asian women. We hypothesized that adding anthropometry to HbA1c would have stronger discriminative ability over HbA1c alone in detecting concurrent dysglycemia. This could have implications for the provision of preconception care by improving our ability to diagnose dysglycemia and optimize preconception health.

## Methods

### Population

The Singapore PREconception Study of long-Term maternal and child Outcomes (S-PRESTO) (ClinicalTrials.gov identifier: NCT03531658) is an ongoing mother-offspring cohort that aims to examine the impact of women’s preconception health, nutritional status, and mental state on their up-coming pregnancy and offspring health outcomes. Briefly, we recruited non-pregnant women (aged 18−45 years) actively trying to conceive from the community (social media, radio adverts, door to door leaflets, posters in public places, general practices) and at Singapore’s largest public maternity unit (KK Women’s and Children’s Hospital). In 2015–2018, 1033 women fulfilling the following eligibility criteria were recruited: intending to reside in Singapore for the next five years; from the three major ethnic groups of Singapore ─ Chinese, Malay, Indian, or a combination of these; and wishing to conceive within one year from recruitment. We excluded women with known pre-existing Type 1 or Type 2 diabetes mellitus prior to study recruitment (Supplementary Table S1). Women were also excluded if they were on oral or implanted contraception, assisted fertility treatment (except clomiphene or letrozole), systemic steroids, anticonvulsants, HIV or Hepatitis B or C medication in the past one month. None of the women were on metformin at the time of enrolment. Every participant had a negative urinary pregnancy test at enrolment. The SingHealth Centralized Institutional Review Board granted ethical approval (reference 2014/692/D), written informed consent was obtained from all women. All methods were carried out in accordance with relevant guidelines and regulations.

### Anthropometric measurements

Anthropometric indices were obtained by trained staff at recruitment using standardized protocols^20^. Weight and height were recorded to the nearest 0.1 kg and 0.1 cm respectively using calibrated scales (SECA 803 Weighing Scale; SECA 213 Stadiometer; SECA, Hamburg, Germany). Waist and hip circumferences were measured with a non-stretchable measuring tape (Seca 212; SECA, Hamburg, Germany). ABSI was defined by: WC/(BMI^2/3^ × height^1/2^)^18^. Four-site skinfold thicknesses (triceps, biceps, subscapular, suprailiac) were measured to the nearest 0.2 mm using skinfold calipers (Holtain Ltd, Crymych, UK) on the right side of the body. For reliability, weight, height, waist and hip circumferences were taken in duplicates, while skinfold measurements were taken in triplicates, and respective measurements were averaged. Anthropometric training and standardization sessions were conducted every three months. The inter-observer technical error of measurement (TE) and coefficient of variation (CV)^21^ are shown in Supplementary Table S2.

### Glucose tolerance tests and HbA1c

Participants underwent a 75-gram OGTT after an overnight fast at recruitment. Venous fasting plasma glucose (FPG), 2-hour plasma glucose (2hPG) and HbA1c were measured (Abbott Architect Plus c8000, Wiesbaden, Germany). Pre-diabetes is defined as impaired fasting glucose (IFG): FPG 6.1–6.9 mmol/L with 2hPG < 11.1 mmol/L, or impaired glucose tolerance (IGT): FPG < 6.1 mmol/L with 2hPG ≥ 7.8 and <11.1 mmol/L. Diabetes is defined as: FPG ≥ 7.0 mmol/L or 2hPG ≥ 11.1 mmol/L^9^. Participants with IFG, IGT or diabetes were collectively grouped as dysglycemia.

### Covariates

Data on: (i) socio-demographics (age, self-reported ethnicity, educational level, smoking history, current employment status, shift work), and (ii) clinical characteristics (menstrual cycle regularity [irregular cycle was defined as having a variation of more than 5 days between periods in the last 6 months], parity, previous history of gestational diabetes mellitus [GDM], history of hypertension, and family history of diabetes in a first degree relative) were gathered at recruitment through interviewer-administered questionnaires.

### Statistical analyses

We performed a cross-sectional analysis of the baseline preconception data from the S-PRESTO cohort. Participants with data for at least one anthropometric or HbA1c measurement as well as complete information on relevant covariates and glucose measures were included (Supplementary Fig. S1). Differences between women with and without dysglycemia were tested using two-sample t-tests for continuous variables and chi-square tests for categorical variables. We standardized all anthropometry and HbA1c to z-scores within the cohort to allow direct comparisons of effect estimates.

Using multiple linear and logistic regression analysis respectively for continuous and categorical outcome variables, we compared each anthropometry and HbA1c to glucose outcomes (FPG, 2hPG, dysglycemia). We adjusted for potential confounders that were significantly related to both exposure and outcome in our population (Analysis 1; educational level, menstrual cycle regularity), and based on published literature (Analysis 2; the aforementioned factors, with addition of age, ethnicity, smoking history, shift work, parity, GDM history, family history of diabetes)^22–24^. The variance in glucose outcomes was assessed using adjusted R^2^ values.

The sensitivity, specificity, and area under receiver-operating characteristic (AUROC) were calculated to evaluate the utility of anthropometry and/or HbA1c in detecting dysglycemia. The performance of each model was compared, with and without history (History: age, ethnicity, educational level, smoking history, shift work, menstrual cycle regularity, parity, GDM history, and family history of diabetes). Continuous values of anthropometry and HbA1c were evaluated first, followed by categorical variables based on clinically-recommended thresholds associated with increased metabolic risks, as would normally be done in clinical practice. These were BMI (≥23 kg/m^2^ and ≥25 kg/m^2^ according to Asian and international thresholds for overweight, respectively)^25^, the 90^th^ percentiles for WHtR and total skinfolds, and HbA1c ≥ 5.7% for pre-diabetes (suggested by the American Diabetes Association)^9^. For categorical data analyses, age as a confounder was dichotomized based on the population’s median age (>30 years). The AUROC of each model was compared with several models as our references: (i) history, (ii) HbA1c, and (iii) BMI. A test of equality was used to test for statistical differences between AUROC values.

To illustrate the clinical utility of different combinations of anthropometry with HbA1c ≥ 5.7 as screening tests, we calculated the positive and negative predictive values (PPV and NPV respectively), the numbers of women identified as requiring OGTT, and the number of detected and missed cases with dysglycemia.

As sensitivity analyses, we excluded women with a previous history of GDM, selected only four commonly considered risk factors as covariates (age, ethnicity, GDM history, family history of diabetes), and evaluated the different thresholds of anthropometry and HbA1c for detecting dysglycemia. The optimal cut-offs of BMI (computed at 0.5 unit increments, ranging from 23 − 27.5 kg/m^2^ characterizing overweight in Asians; followed by 0.1 unit increments within the identified optimal range from 23 − 24 kg/m^2^), WHtR and total skinfolds (95^th^ and 99^th^ percentiles) and HbA1c (computed at 0.1 unit increments, ranging from 5.0 − 5.7%) which yielded the highest AUROC were identified and used in the model-comparison analysis. Additional sensitivity analyses were conducted with alternative established cut-offs of BMI ≥ 27.5 kg/m^2^ for obese in Asians^25^ and HbA1c ≥ 6.0%^26^. Analyses were performed using Stata 15.1 software (StataCorp LP, TX). Statistical significance was considered at P < 0.05.

## Results

A total of 971 women had at least one anthropometric measure with complete FPG and 2hPG results (Supplementary Fig. S1), and 946 had both complete OGTT results and HbA1c data available for relevant AUROC analysis. There were no significant differences in the characteristics of excluded and included participants, except for a greater proportion of women with a higher level of tertiary education in the included group (Supplementary Table S3). Of those included, 106 women (10.9%) had dysglycemia. The median (interquartile range, IQR) of FPG, 2hPG and HbA1c were 4.7 mmol/L (4.5–5.0), 5.6 mmol/L (4.8–6.2) and 5.1% (4.9–5.2) respectively in women with normal glucose tolerance; and 5.1 mmol/L (4.7–5.6), 8.8 mmol/L (8.2–10.1) and 5.3% (5.1–5.6) respectively in women with dysglycemia (Supplementary Figs. S2 and S3). Women with dysglycemia were less likely to have attained tertiary education, more likely to have had menstrual cycle irregularity and previous GDM than women with normal glucose tolerance (Table 1). Women with dysglycemia also exhibited significantly higher values for all anthropometric measures compared to women with normal glucose tolerance, except for ABSI (Supplementary Table S4).

**Table 1:** Demographics and clinical characteristics of all women by glycemic status.

		All (%)^†^ (n = 971)	Normal Glucose Tolerance (n = 865)	Dysglycemia (n = 106)	p-value
***Demographics***^‡^					
**Age (year)**^§^	971 (100.0)	30.8 ± 3.7	30.8 ± 4.0		0.97
**Ethnicity**	971 (100.0)				0.21
Chinese			630 (72.8)	68 (64.2)	
Malay			130 (15.0)	23 (21.7)	
Indian			75 (8.7)	12 (11.3)	
Mixed			30 (3.5)	3 (2.8)	
**Educational level**	971 (100.0)				<0.01
No/Primary/Secondary			98 (11.3)	16 (15.1)	
Post-secondary			202 (23.4)	41 (38.7)	
Tertiary			565 (65.3)	49 (46.2)	
**Smoking history**	971 (100.0)				0.55
Never			775 (89.6)	93 (87.7)	
Previous smoker			51 (5.9)	9 (8.5)	
Current smoker			39 (4.5)	4 (3.8)	
**Employment status**	971 (100.0)				0.37
Unemployed			119 (13.8)	18 (17.0)	
Employed			746 (86.2)	88 (83.0)	
**Shift work**	971 (100.0)				0.41
No/Unemployed			781 (90.3)	93 (87.7)	
Yes			84 (9.7)	13 (12.3)	
***Clinical characteristics***^‡^					
**Menstrual cycle regularity**	971 (100.0)				<0.01
Regular			576 (66.6)	53 (50.0)	
Irregular			289 (33.4)	53 (50.0)	
**Parity**	971 (100.0)				0.95
Nulliparous			560 (64.7)	67 (63.2)	
Primiparous			232 (26.8)	30 (28.3)	
Multiparous			73 (8.4)	9 (8.5)	
**Previous history of GDM**^¶^	490 (50.4)				0.06^¶^
No			411 (95.8)	55 (90.2)	
Yes			18 (4.2)	6 (9.8)	
**History of hypertension**	971 (100.0)				0.08
No			861 (99.5)	104 (98.1)	
Yes			4 (0.5)	2 (1.9)	
**Family history of diabetes**	971 (100.0)				0.33
No			611 (70.6)	70 (66.0)	
Yes			254 (29.4)	36 (34.0)	

^†^Total sample size (n) is not always 971 due to the missing values.

^‡ʹ^ (%).

^§^Mean ± SD.

^¶^Excluding nulliparous women.

GDM, Gestational diabetes mellitus.

To select the most appropriate anthropometric measures for use in screening, we compared the association of each measure, alongside HbA1c, with glucose outcomes (Table 2). We observed significant positive associations of each standardized anthropometric measure and HbA1c with FPG, 2hPG and dysglycemia. Of all the anthropometric measures, BMI, WHtR (amongst the abdominal adiposity indices) and total skinfolds (amongst the skinfold measures) were most strongly associated with FPG, 2hPG, and dysglycemia. ABSI was not significantly associated with any glycemic outcomes, yielding the smallest R^2^ of all anthropometric indices and therefore, excluded from subsequent analyses.

**Table 2:** Associations of anthropometric and HbA1c z-scores with glucose outcomes in reproductive age women.

z-scores	FPG			2hPG			Dysglycemia		
	Analysis 1 β (95% CI)	Analysis 2 β (95% CI)	Adj R^2^ (%)	Analysis 1 β (95% CI)	Analysis 2 β (95% CI)	Adj R^2^ (%)	Analysis 1 OR (95% CI)	Analysis 2 OR (95% CI)	Adj R^2^ (%)
BMI	0.18 (0.13, 0.23)	0.18 (0.13, 0.23)	9.6	0.53 (0.40, 0.66)	0.51 (0.37, 0.66)	10.5	1.60 (1.32–1.93)	1.62 (1.32–1.99)	8.1
Waist Circumference	0.16 (0.11, 0.21)	0.15 (0.10, 0.20)	8.3	0.47 (0.33, 0.60)	0.45 (0.31, 0.59)	9.5	1.55 (1.27–1.89)	1.59 (1.28–1.98)	7.5
Hip Circumference	0.14 (0.09, 0.19)	0.13 (0.08, 0.18)	7.6	0.35 (0.21, 0.48)	0.32 (0.18, 0.46)	7.7	1.34 (1.10–1.63)	1.32 (1.06–1.63)	5.9
Waist-to-hip ratio	0.09 (0.04, 0.13)	0.08 (0.03, 0.13)	6.0	0.35 (0.22, 0.48)	0.33 (0.20, 0.46)	8.1	1.55 (1.25–1.92)	1.60 (1.28–2.00)	7.5
Waist-to-height ratio	0.17 (0.12, 0.22)	0.17 (0.11, 0.22)	8.8	0.53 (0.40, 0.67)	0.53 (0.38, 0.67)	10.6	1.66 (1.36–2.03)	1.74 (1.39–2.17)	8.4
ABSI	−0.02 (−0.07, 0.03)	−0.03 (−0.07, 0.02)	5.1	−0.02 (−0.14, 0.11)	−0.03 (−0.16, 0.1)	5.8	0.95 (0.77–1.17)	0.96 (0.77–1.18)	5.0
Peripheral skinfolds	0.17 (0.12, 0.21)	0.15 (0.11, 0.2)	8.7	0.57 (0.44, 0.70)	0.54 (0.40, 0.68)	11.5	1.80 (1.47–2.20)	1.82 (1.46–2.27)	9.3
Truncal skinfolds	0.17 (0.12, 0.21)	0.15 (0.10, 0.2)	8.4	0.57 (0.44, 0.70)	0.53 (0.39, 0.66)	11.8	1.94 (1.57–2.40)	1.95 (1.56–2.45)	10.5
Total skinfolds	0.17 (0.12, 0.22)	0.16 (0.11, 0.2)	8.5	0.59 (0.46, 0.72)	0.56 (0.42, 0.69)	12.3	1.97 (1.59–2.44)	2.02 (1.60–2.55)	10.8
Subscapular-to-triceps ratio	0.11 (0.07, 0.16)	0.12 (0.07, 0.16)	7.1	0.34 (0.21, 0.47)	0.34 (0.21, 0.47)	8.8	1.50 (1.23–1.83)	1.53 (1.25–1.87)	7.6
HbA1c	0.59 (0.56, 0.62)	0.58 (0.55, 0.61)	62.5	1.41 (1.31, 1.51)	1.39 (1.29, 1.49)	47.7	3.84 (2.69–5.47)	4.09 (2.81–5.94)	18.2

Regression estimates and odds ratios reflect a 1-SD increase in each index per mmol/L increment in glucose and with dysglycemia compared with normal glucose tolerance, respectively.

Analysis 1: Adjusted for educational level and menstrual cycle regularity.

Analysis 2: Adjusted for all covariates in Analysis 1; age, ethnicity, smoking history, shift work, parity, GDM history and family history of diabetes.

Adjusted R^2^: Explained variance for Analysis 2.

ABSI, A Body Shape Index; BMI, body mass index; CI, confidence interval; FPG, Fasting plasma glucose; HbA1c, glycated hemoglobin; OR, odds ratio; 2hPG, 2-hour plasma glucose.

**Figure 1:**
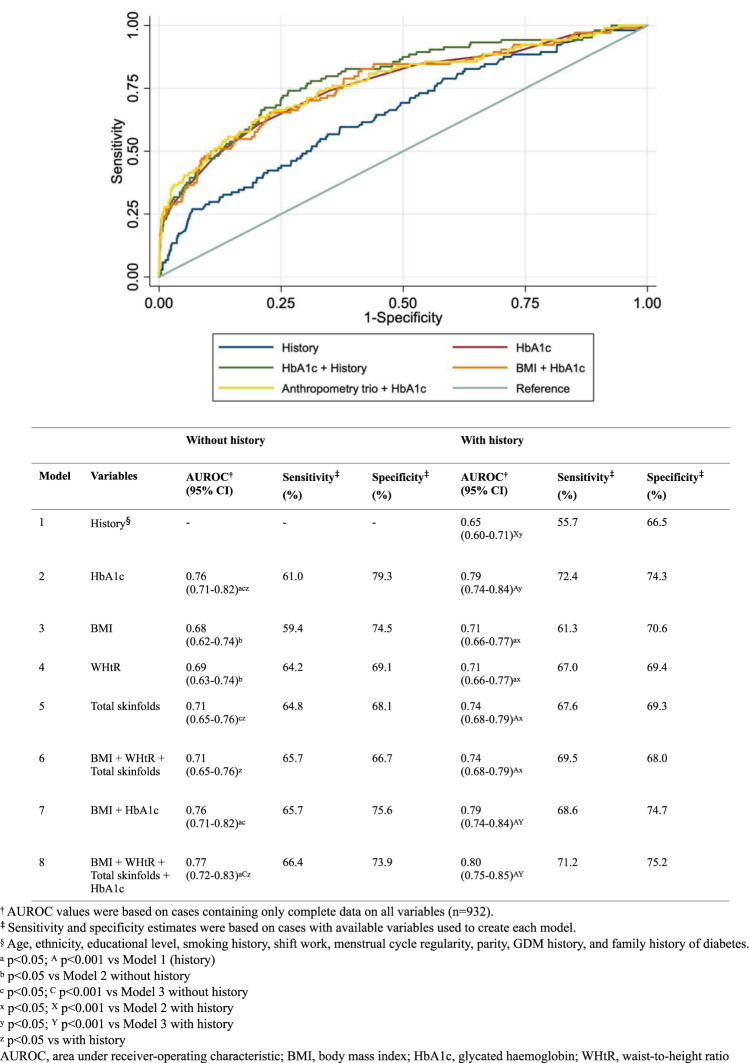
Continuous measures of anthropometry and HbA1c – The AUROC, sensitivity and specificity in detecting dysglycemia without and with adjustment for history.

As continuous variables, the AUROC of HbA1c alone was higher than each of the anthropometric measures (BMI, WHtR, total skinfolds) (Fig. 1). Without adjustment for history, adding this anthropometry trio (AUROC 0.77) or simply BMI (0.76) to HbA1c did not improve the AUROC of HbA1c alone (0.76). With adjustment for history, the AUROC of HbA1c alone was improved as compared to HbA1c without history (0.79 vs 0.76, p = 0.041). Similarly, the AUROC for the combination of anthropometry trio and HbA1c was higher with than without history (0.80 vs 0.77, p = 0.03). With history, each of the three anthropometric measures had similar sensitivities (61.3–67.6%), specificities (69.3–70.6%) and AUROC values (0.71–0.74), and as a trio, did not outperform HbA1c alone (0.74 vs 0.79, p = 0.02).

**Figure 2:**
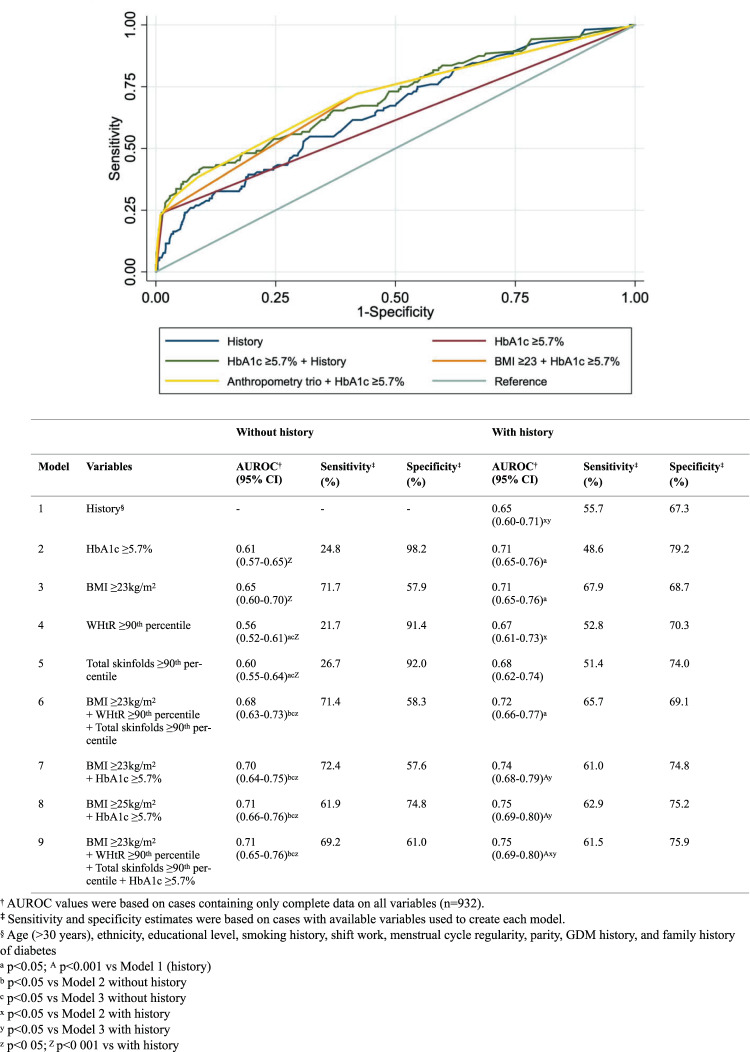
Categorical measures of anthropometry and HbA1c – The AUROC, sensitivity and specificity in detecting dysglycemia without and with adjustment for history.

However, in clinical practice, screening tests are interpreted using predefined validated thresholds rather than as continuous variables. We thus assessed the clinically-relevant thresholds of BMI ≥ 23 kg/m^2^, WHtR ≥  90^th^ percentile, total skinfolds ≥90^th^ percentile, and HbA1c ≥ 5.7% (Fig. 2). Without adjustment for history, the AUROC for HbA1c ≥ 5.7% alone (0.61) was similar to each of the three anthropometric measures (0.56–0.65). Adding BMI ≥ 23 kg/m^2^ to HbA1c ≥ 5.7% significantly improved the sensitivity (24.8% vs 72.4%) and AUROC (0.61 vs 0.70, p = 0.0002) of HbA1c ≥ 5.7% alone when history was not considered. The AUROC of BMI ≥ 23 kg/m^2^ alone (0.65) was higher than that of the ≥90^th^ percentiles of WHtR (0.56, p = 0.001) and total skinfolds (0.60, p = 0.03) but not as high as that of the anthropometry trio (0.68, p = 0.003). However, adding the anthropometry trio to HbA1c ≥ 5.7% (0.71) fared similarly to simply adding BMI ≥ 23 kg/m^2^ to HbA1c ≥ 5.7% (0.70). Using the international BMI cut-off of ≥25 kg/m^2^ (representing overweight/obese) to HbA1c ≥ 5.7% yielded similar AUROC (0.70 vs 0.71) as using the Asian cut-off of BMI ≥ 23 kg/m^2^, but the sensitivity was lower with the BMI ≥ 25 kg/m^2^ combination (61.9% vs 72.4%). Overall, adding history improved the AUROC of HbA1c ≥ 5.7% as well as each anthropometric measure and in various combinations. In sensitivity analyses, incorporating only the more commonly considered risk factors of age, ethnicity, previous history of GDM and family history of diabetes, AUROC results were largely similar to when the full set of covariates was used (Supplementary Table S5).

Table 3 illustrates the use of several options for first-stage screening in women with both BMI and HbA1c measures. Despite achieving the highest PPV among all, HbA1c ≥ 5.7% on its own yielded the lowest sensitivity, with about 75% of all dysglycemia cases remaining undiagnosed. Adding BMI ≥ 23 kg/m^2^ to HbA1c ≥ 5.7% would increase the proportion of women detected with dysglycemia by threefold. Although many more women would still require an OGTT, this would be less than half the number compared with a universal OGTT strategy. Adding further anthropometry measures to HbA1c ≥ 5.7% did not appreciably reduce the numbers requiring OGTT as compared to adding BMI ≥ 23 kg/m^2^ alone. Increasing the BMI threshold to ≥25 kg/m^2^ or adding history to BMI ≥ 23 kg/m^2^ in conjunction with HbA1c ≥ 5.7% performed similarly to each other; both reducing the numbers of women requiring an OGTT to less than one-third, but missing almost 40% of dysglycemia cases. As simply having a history of GDM would warrant a preconception OGTT, sensitivity analyses were performed by excluding women with a previous history of GDM (n = 24), which showed largely similar results (Supplementary Table S6).

**Table 3:** Clinical utility of different strategies in detecting dysglycemia preconception among 946 Asian women who provided both BMI and HbA1C measures (105 out of 946 women with HbA1c measure had dysglycemia).

		Positive predictive value (%)	Negative predictive value (%)	Number to proceed to OGTT (% of all screened)	Number of dysglycemia identified (% of all dysglycemia)	Number of dysglycemia cases missed (% of all dysglycemia)
1.	HbA1c ≥ 5.7%	63.4	91.3	41 (4.3%)	26 (24.8%)	79 (75.2%)
2.	BMI ≥ 23 kg/m^2^ + HbA1c ≥ 5.7%	17.6	94.4	433 (45.8%)	76 (72.4%)	29 (27.6%)
3.	BMI ≥ 23 kg/m^2^ + WHtR ≥90th percentile + Total skinfolds ≥90th percentile + HbA1c ≥ 5.7%^†^	18.2	94.0	395 (42.4%)	72 (69.2%)	32 (30.8%)
4.	BMI ≥ 25 kg/m^2^ + HbA1c ≥ 5.7%	23.5	94.0	277 (29.3%)	65 (61.9%)	40 (38.1%)
5.	BMI ≥ 23 kg/m^2^ + HbA1c ≥ 5.7% + History^‡^	23.2	93.9	276 (29.2%)	64 (61.0%)	41 (39.0%)

^†^n = 932 provided all measurements (104 out of 932 women with dysglycemia). ^‡^History variables included: Age (>30 years), ethnicity, educational level, smoking history, shift work, menstrual cycle regularity, parity, GDM history, and family history of diabetes.

Different cut-offs for BMI, WHtR, total skinfolds, and HbA1c in detecting dysglycemia are presented in Supplementary Table S7. Since the performance of WHtR and total skinfolds as categorical variables were generally poor, these measures were not explored further. Using various combinations of anthropometric thresholds and HbA1c yielded similar findings as the commonly used clinical thresholds (Supplementary Table S8). Further sensitivity analyses showed that BMI ≥ 27.5 kg/m^2^ for defining obesity in Asians and HbA1c ≥ 6.0% also yielded qualitatively similar associations to those of the primary results.

## Discussion

In this multi-ethnic Singaporean population of Asian women trying to conceive, using BMI ≥ 23 kg/m^2^ and HbA1c ≥ 5.7% for screening was better than HbA1c ≥ 5.7% alone in detecting dysglycemia. This strategy of adding BMI to HbA1c reduced the proportion of undetected cases by almost two-thirds when history was not considered in a screening algorithm, as is the case in standard clinical practice. There was limited additional value in the use of other anthropometric measures in place of BMI together with HbA1c.

The association of anthropometry, a surrogate marker of insulin resistance, with diabetes in adult populations has been equivocal. Consistent with our results, two meta-analyses found that BMI, WHtR, and WC were equally discriminative in detecting incident diabetes^27^. A Canadian study found skinfold thickness in combination with WC and BMI were strong indicators of glucose intolerance^19^, just as we found a combination of anthropometric measures (BMI, WHtR, total skinfolds) outperformed BMI alone. Other studies utilizing a longitudinal rather than a cross-sectional design, however, reported changes in WC or WHtR over time as a stronger discriminator over BMI. Our finding of no association between ABSI and dysglycemia was in contrast to those of a previous meta-analysis^28^. Given that ABSI was originally developed using data from a US population comprising predominantly Whites, Blacks, and Hispanics, ethnically different body shapes and fat distribution may explain the discrepancy^29^.

BMI is the most widely used anthropometric measure as it has negligible costs, is easy to measure and highly reproducible. Conversely, measurements of WC, WHtR, and skinfolds are limited by the lack of technical standardization and reproducibility in clinical practice. In our study, even with a high level of training, coefficient of variation in skinfold measurements was still larger relative to that of weight and height (Supplementary Table S2). As we found marginal advantage in using anthropometric combinations with HbA1c, and the potential for measurement errors, the cost-benefit of applying such anthropometric measurements is unlikely to be favourable and remains to be evaluated.

Given the low patient acceptability and challenges of universal screening with OGTT for dysglycemia during preconception, we propose a two-stage screening strategy. Our results show HbA1c remains an important tool in the first stage to identify high risk women who warrant further testing in the second stage with a diagnostic OGTT. Using HbA1c together with anthropometry as continuous variables increased statistical power and provided the best results in detecting dysglycemia but is difficult to implement in clinical practice without applying highly sophisticated mathematical algorithms alongside population-specific clinical factors. Thus, using pre-defined clinical thresholds of measurable parameters remains the main stay in clinical management.

Relying solely on HbA1c ≥ 5.7% resulted in the bulk of dysglycemia cases remaining undetected in our population. With the highest sensitivity, a combination of BMI ≥ 23 kg/m^2^ and HbA1c ≥ 5.7% was the best first-stage screening strategy to capture as many dysglycemia cases as possible, but it also had the lowest PPV. This has to be balanced against patient and healthcare burdens. Although disliked by women, given the relative safety and low expense of an OGTT as a second line test which is already used in routine clinical practice, this is an acceptable strategy to adopt. Such a strategy would also halve the proportion of women offered an OGTT compared to a single-step universal OGTT screening strategy. However, 27.6% of preconception dysglycemia cases would still be missed. Attempts to optimize the PPV by increasing the BMI threshold or incorporating history merely reduced the number of cases requiring OGTT by 15% but still missed a further 10% of dysglycemia cases compared with the BMI ≥ 23 kg/m^2^ and HbA1c ≥ 5.7% combination, and therefore of no substantial increased benefit.

Overall, incorporating clinical history improved the discriminative abilities (AUROC values) of anthropometry and HbA1c in detecting dysglycemia. However, in any strategy that included BMI ≥ 23 kg/m^2^, adding history yielded a lower sensitivity than the corresponding model without history. Furthermore, history information is rarely used systematically in this context. Thus efforts to develop more elaborate systematic risk scoring algorithms including clinical history, anthropometry and HbA1c may not be of further substantial benefit to improve the detection of women with dysglycemia. At present, universal advice on diet and lifestyle, including folic acid supplementation, is commonly given to women during preconception to optimize pregnancy outcome^30^. The cost-effectiveness of screening for preconception dysglycemia to provide more targeted therapies in reducing hyperglycemia-related pregnancy adversity is unclear, even in a population like Singapore with a high GDM rate of around 20%^31^.

Previous history of GDM, as an indication for a preconception OGTT in many guidelines, is not applicable to nulliparous women which comprise a significant proportion in many developed countries including Singapore, where women prefer smaller families. Furthermore, in settings where universal screening for GDM is not practised, many GDM cases could be missed in previous pregnancies. Hence, there need be pragmatic risk assessments for the whole preconception population, whilst recognizing that preconception dysglycemia screening does not negate the need for universal GDM screening during pregnancy.

Strengths of our study include the analysis of a large Asian cohort recruited from a mixture of low- and high-risk settings (community and a tertiary maternity unit) comprising three major ethnic groups in Singapore, with all women trying to conceive and phenotyped using  comprehensive anthropometric measurements. A large set of potentially confounding variables and history were adjusted for in our analyses. However, as this was a cross-sectional study, we could not exclude the possibility that some women might have attempted weight loss, which could affect their anthropometric measures prior to significant changes in their metabolism as reflected by glucose concentrations and HbA1C measures. Another limitation is that women were not assessed for polycystic ovary syndrome, a risk factor for dysglycemia.

In conclusion, HbA1c in combination with BMI is a fairly sensitive and pragmatic approach that may be used in routine clinical care as first-line screening tests for dysglycemia in reproductive age Asian women trying to conceive. Further longitudinal research and larger epidemiological studies are warranted to assess if other combinations of anthropometry and changes in their corresponding thresholds or other metabolic-related measures could further improve the detection rate of dysglycemia preconception, and better still, if they could also be useful for predicting future GDM.

## Supplementary information

Supplementary information.

## Data Availability

Data are available upon request to the S-PRESTO team.

## References

[1] NCD Risk Factor Collaboration (2016). Worldwide trends in diabetes since 1980: a pooled analysis of 751 population-based studies with 4.4 million participants. Lancet..

[2] Beagley J, Guariguata L, Weil C, Motala AA (2014). Global estimates of undiagnosed diabetes in adults. Diabetes Res Clin Pract..

[3] Reece EA, Leguizamón G, Wiznitzer A (2009). Gestational diabetes: the need for a common ground. Lancet..

[4] Dabelea D (2000). Intrauterine exposure to diabetes conveys risks for type 2 diabetes and obesity: a study of discordant sibships. Diabetes..

[5] Ma RCW, Popkin BM (2017). Intergenerational diabetes and obesity-A cycle to break?. PLoS Med..

[6] Diabetes care and research in Europe (1990). The St. Vincent Declaration. Diabet Med..

[7] Fleming TP (2018). Origins of lifetime health around the time of conception: causes and consequences. Lancet..

[8] Bearak J, Popinchalk A, Alkema L, Sedgh G (2018). Global, regional, and subregional trends in unintended pregnancy and its outcomes from 1990 to 2014: estimates from a Bayesian hierarchical model. Lancet Glob Health..

[9] World Health Organization. Definition and diagnosis of diabetes mellitus and intermediate hyperglycemia: report of a WHO/IDF consultation. Geneva: World Hearth Organization. (2006).

[10] American Diabetes Association. 2 (2019). Classification and diagnosis of diabetes: standards of medical care in diabetes—2019. Diabetes Care..

[11] Wheeler E (2017). Impact of common genetic determinants of Hemoglobin A1c on type 2 diabetes risk and diagnosis in ancestrally diverse populations: A transethnic genome-wide meta-analysis. PLoS Med..

[12] Fucharoen S, Winichagoon P (2011). Haemoglobinopathies in Southeast Asia. Indian J Med Res..

[13] International Expert Committee (2009). International Expert Committee report on the role of the A1C assay in the diagnosis of diabetes. Diabetes Care..

[14] American Diabetes Association (2012). Standards of medical care in diabetes—2012. Diabetes Care..

[15] Hartwig S (2015). Association of change of anthropometric measurements with incident type 2 diabetes mellitus: a pooled analysis of the prospective population-based CARLA and SHIP cohort studies. Medicine..

[16] Bi X (2015). DXA-measured visceral adipose tissue predicts impaired glucose tolerance and metabolic syndrome in obese Caucasian and African-American women. Eur J Clin Nutr..

[17] Ross R, Fortier L, Hudson R (1996). Separate associations between visceral and subcutaneous adipose tissue distribution, insulin and glucose levels in obese women. Diabetes Care..

[18] Krakauer NY, Krakauer JC (2012). A new body shape index predicts mortality hazard independently of body mass index. PLoS One..

[19] Sievenpiper JL (2001). Simple skinfold-thickness measurements complement conventional anthropometric assessments in predicting glucose tolerance. Am J Clin Nutr..

[20] Hamilton CM (2011). The PhenX Toolkit: get the most from your measures. Am J Epidemiol..

[21] Perini TA, de Oliveira GL, Ornellas JDS, de Oliveira FP (2005). Technical error of measurement in anthropometry. Revista Brasileira de Medicina do Esporte..

[22] Nicholson WK (2006). Parity and risk of type 2 diabetes. Diabetes Care..

[23] Solomon CG (2001). Long or highly irregular menstrual cycles as a marker for risk of type 2 diabetes mellitus. JAMA..

[24] Pan A, Schernhammer ES, Sun Q, Hu FB (2011). Rotating night shift work and risk of type 2 diabetes: two prospective cohort studies in women. PLoS Med..

[25] WHO Expert Consultation (2004). Appropriate body-mass index for Asian populations and its implications for policy and intervention strategies. Lancet..

[26] Selvin E (2010). Glycated hemoglobin, diabetes, and cardiovascular risk in nondiabetic adults. N Engl J Med..

[27] Vazquez G, Duval S, Jacobs DR, Silventoinen K (2007). Comparison of body mass index, waist circumference, and waist/hip ratio in predicting incident diabetes: a meta-analysis. Epidemiol Rev..

[28] Ji M, Zhang S, An R (2018). Effectiveness of A Body Shape Index (ABSI) in predicting chronic diseases and mortality: a systematic review and meta-analysis. Obes Rev..

[29] Lim LL, Tan ATB, Moses K, Rajadhyaksha V, Chan SP (2017). Place of sodium-glucose cotransporter-2 inhibitors in East Asian subjects with type 2 diabetes mellitus: Insights into the management of Asian phenotype. J Diabetes Complications..

[30] World Health Organization. Guideline: daily iron and folic acid supplementation in pregnant women: Geneva: World Health Organization (2012). 23586119

[31] Chong YS (2014). Ethnic differences translate to inadequacy of high-risk screening for gestational diabetes mellitus in an Asian population: a cohort study. BMC Pregnancy Childbirth..

