# Author Correction: Anthropometric measures and HbA1c to detect dysglycemia in young Asian women planning conception: The S-PRESTO cohort

**DOI:** 10.1038/s41598-020-69653-0

**Published:** 2020-07-24

**Authors:** Anne H. Y. Chu, Izzuddin M. Aris, Sharon Ng, See Ling Loy, Jonathan Y. Bernard, Mya Thway Tint, Wen Lun Yuan, Keith M. Godfrey, Jerry Kok Yen Chan, Lynette Pei-Chi Shek, Yap Seng Chong, Kok Hian Tan, Seng Bin Ang, Heng Hao Tan, Bernard S. M. Chern, Fabian Yap, Yung Seng Lee, Ngee Lek, Melvin Khee-Shing Leow, Chin Meng Khoo, Shiao-Yng Chan

**Affiliations:** 1 Singapore Institute for Clinical Sciences (SICS), Agency for Science, Technology and Research (A*STAR), Singapore, Singapore; 2 Department of Obstetrics and Gynaecology, Yong Loo Lin School of Medicine, National University of Singapore, Singapore, Singapore; 3 Division of Chronic Disease Research Across the Lifecourse, Department of Population Medicine, Harvard Medical School and Harvard Pilgrim Health Care Institute, Boston, Massachusetts USA; 4 Department of Reproductive Medicine, KK Women’s and Children Hospital, Singapore, Singapore; 5 Duke-NUS Medical School, Singapore, Singapore; 6 Centre for Research in Epidemiology and Statistics, Inserm, Villejuif, France; 7 Department of Paediatrics, Yong Loo Lin School of Medicine, National University of Singapore, Singapore, Singapore; 8 MRC Lifecourse Epidemiology Unit and NIHR Southampton Biomedical Research Centre, University of Southampton and University Hospital Southampton NHS Foundation Trust, Southampton, UK; 9 Department of Maternal Fetal Medicine, KK Women’s and Children’s Hospital, Singapore, Singapore; 10 Department of Family Medicine Service, KK Women’s and Children’s Hospital, Singapore, Singapore; 11 Division of Obstetrics & Gynaecology, KK Women’s and Children’s Hospital, Singapore, Singapore; 12 Lee Kong Chian School of Medicine, Nanyang Technological University, Singapore, Singapore; 13 Department of Paediatrics, KK Women’s and Children’s Hospital, Singapore, Singapore; 14 Department of Medicine, Yong Loo Lin School of Medicine, National University of Singapore, Singapore, Singapore

Correction to: *Scientific Reports* https://doi.org/10.1038/s41598-020-66147-x, published online 08 June 2020

This Article contains errors in Table 1. In the HTML and PDF versions of this Article, the headers for columns *n* and All have been merged, and some of the characteristics in the column ‘All’ are missing. The correct Table 1 appears below.

**Table 1:** Demographics and clinical characteristics of all women by glycemic status.

	n	All	Normal Glucose Tolerance (n = 865)	Dysglycemia (n = 106)	p-value*
**Demographics**^†^					
**Age (year)**^‡^	971	30.8 ± 3.7	30.8 ± 3.7	30.8 ± 4.0	0.97
**Ethnicity**	971				0.21
Chinese		698 (71.9)	630 (72.8)	68 (64.2)	
Malay		153 (15.7)	130 (15.0)	23 (21.7)	
Indian		87 (9.0)	75 (8.7)	12 (11.3)	
Mixed		33 (3.4)	30 (3.5)	3 (2.8)	
**Educational level**	971				< 0.01
No/Primary/Secondary		114 (11.7)	98 (11.3)	16 (15.1)	
Post-secondary		243 (25.0)	202 (23.4)	41 (38.7)	
Tertiary		614 (63.2)	565 (65.3)	49 (46.2)	
**Smoking history**	971				0.55
Never		868 (89.4)	775 (89.6)	93 (87.7)	
Previous smoker		60 (6.2)	51 (5.9)	9 (8.5)	
Current smoker		43 (4.4)	39 (4.5)	4 (3.8)	
**Employment status**	971				0.37
Unemployed		137 (14.1)	119 (13.8)	18 (17.0)	
Employed		834 (85.9)	746 (86.2)	88 (83.0)	
**Shift work**	971				0.41
No/Unemployed		874 (90.0)	781 (90.3)	93 (87.7)	
Yes		97 (10.0)	84 (9.7)	13 (12.3)	
**Clinical characteristics**^†^					
**Menstrual cycle regularity**	971				< 0.01
Regular		629 (64.8)	576 (66.6)	53 (50.0)	
Irregular		342 (35.2)	289 (33.4)	53 (50.0)	
**Parity**	971				0.95
Nulliparous		627 (64.6)	560 (64.7)	67 (63.2)	
Primiparous		262 (27.0)	232 (26.8)	30 (28.3)	
Multiparous		82 (8.4)	73 (8.4)	9 (8.5)	
**Previous history of GDM**^§^	490				0.06
No		466 (95.1)	411 (95.8)	55 (90.2)	
Yes		24 (4.9)	18 (4.2)	6 (9.8)	
**History of hypertension**	971				0.08
No		965 (99.4)	861 (99.5)	104 (98.1)	
Yes		6 (0.6)	4 (0.5)	2 (1.9)	
**Family history of diabetes**	971				0.33
No		681 (70.1)	611 (70.6)	70 (66.0)	
Yes		290 (29.9)	254 (29.4)	36 (34.0)	

^†^n (%).

^‡^Mean ± SD.

^§^Excluding nulliparous women.

*Significant differences between women with normal glucose tolerance and women without dysglycemia.

GDM, Gestational diabetes mellitus.

Additionally, there is a citation error in the Methods section under subheading ‘Statistical analyses’, where,

“These were BMI (≥ 23 kg/m^2^ and ≥ 25 kg/m^2^ according to Asian and international thresholds for overweight, respectively)^25^, the 90^th^ percentiles for WHtR and total skinfolds, and HbA1c ≥ 5.7% for pre-diabetes (suggested by the American Diabetes Association)^9^.”

should read:

“These were BMI (≥ 23 kg/m^2^ and ≥ 25 kg/m^2^ according to Asian and international thresholds for overweight, respectively)^25^, the 90^th^ percentiles for WHtR and total skinfolds, and HbA1c ≥ 5.7% for pre-diabetes (suggested by the American Diabetes Association)^10^.”

There is an error in the Discussion section where,

“Consistent with our results, two meta-analyses found that BMI, WHtR, and WC were equally discriminative in detecting incident diabetes^27^.”

should read:

“Consistent with our results, a meta-analysis found that BMI, WHtR, and WC were equally discriminative in detecting incident diabetes^27^.”

