# Supplementary material for: Anthropometric measures and HbA1c to detect dysglycemia in young Asian women planning conception: The S-PRESTO cohort
Source: Sci Rep. 2020 Jun 8;10:9228. doi: 10.1038/s41598-020-66147-x (PMC7280215; doi:10.1038/s41598-020-66147-x)
Supplement: Supplementary file 1 — Supplementary information. [file 41598_2020_66147_MOESM1_ESM.pdf]

Anthropometric measures and HbA1c to detect dysglycemia in young Asian women planning conception: The S-PRESTO cohort

Anne HY Chu[1], Izzuddin M Aris[1,2,3], Sharon Ng[2], See Ling Loy[4,5], Jonathan Y Bernard[1,6], Mya Thway Tint[1,2], Wen Lun Yuan[7], Keith M Godfrey[8], Jerry Kok Yen Chan[4,5], Lynette Pei-Chi Shek[1,7], Yap Seng Chong[1,2], Kok Hian Tan[5,9], Seng Bin Ang[10], Heng Hao Tan[4,5], Bernard SM Chern[5,11], Fabian Yap[12,13], Yung Seng Lee[1,7], Ngee Lek[5,13], Melvin Khee-Shing Leow[1,5], Chin Meng Khoo[14], Shiao-Yng Chan[1,2*]

[1]Singapore Institute for Clinical Sciences (SICS), Agency for Science, Technology and Research (A*STAR), Singapore
[2]Department of Obstetrics and Gynaecology, Yong Loo Lin School of Medicine, National University of Singapore, Singapore
[3]Division of Chronic Disease Research Across the Lifecourse, Department of Population Medicine, Harvard Medical School and Harvard Pilgrim Health Care Institute, Boston, Massachusetts, USA
[4]Department of Reproductive Medicine, KK Women's and Children Hospital, Singapore
[5]Duke-NUS Medical School, Singapore
[6]Centre for Research in Epidemiology and Statistics, Inserm, Villejuif, France
[7]Department of Paediatrics, Yong Loo Lin School of Medicine, National University of Singapore, Singapore
[8]MRC Lifecourse Epidemiology Unit and NIHR Southampton Biomedical Research Centre, University of Southampton and University Hospital Southampton NHS Foundation Trust, Southampton, UK
[9]Department of Maternal Fetal Medicine, KK Women's and Children's Hospital, Singapore
[10]Department of Family Medicine Service, KK Women's and Children's Hospital, Singapore
[11]Division of Obstetrics & Gynaecology, KK Women's and Children's Hospital, Singapore
[12]Lee Kong Chian School of Medicine, Nanyang Technological University, Singapore
[13]Department of Paediatrics, KK Women's and Children's Hospital, Singapore
[14]Department of Medicine, Yong Loo Lin School of Medicine, National University of Singapore, Singapore

Address reprints and correspondence to:
*Shiao-Yng Chan. Department of Obstetrics and Gynaecology, Yong Loo Lin School of Medicine, National University of Singapore. NUHS Tower Block Level 12, 1E Kent Ridge Road, Singapore 119228. Email: obgchan@nus.edu.sg

Supplementary materials

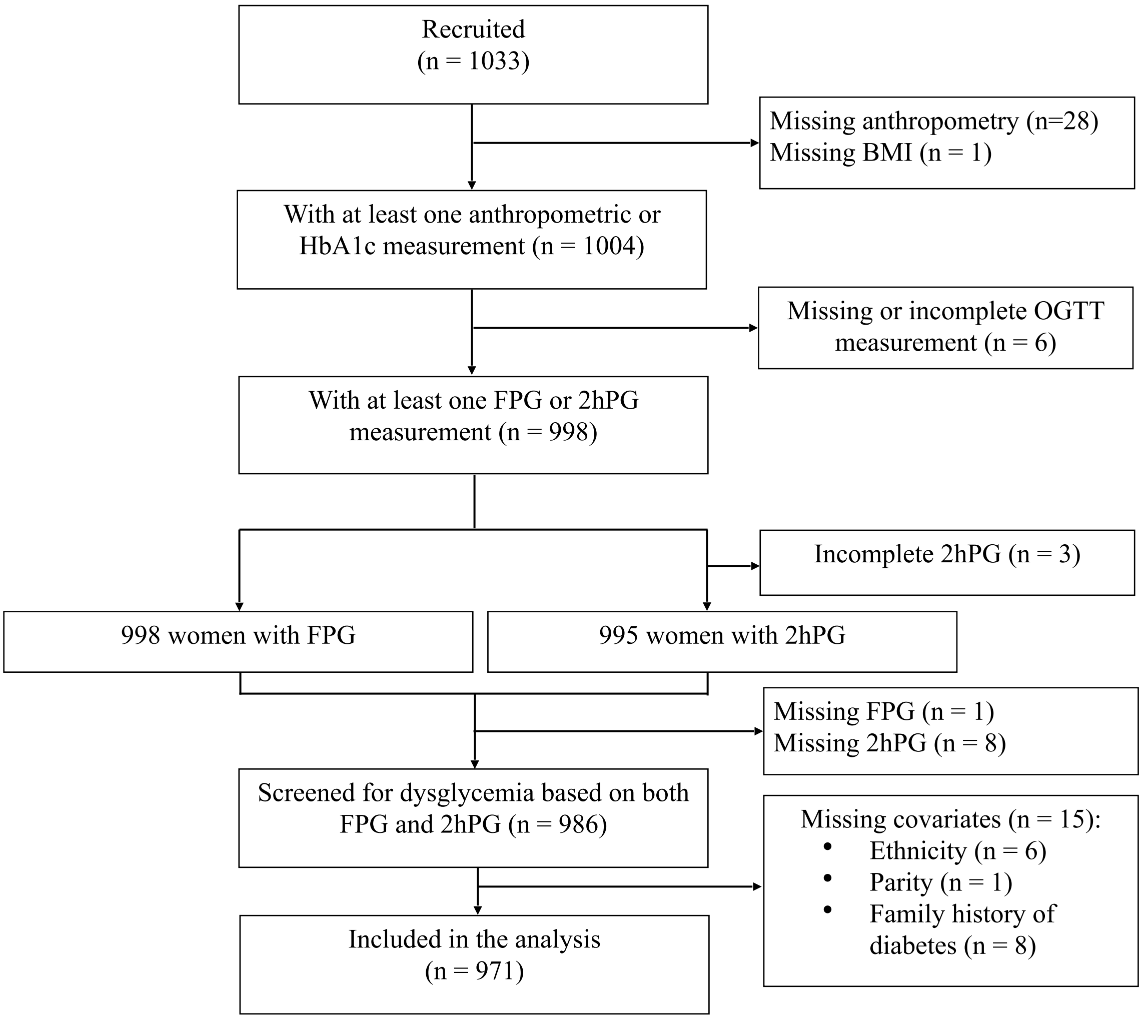

Figure S1: Study flowchart

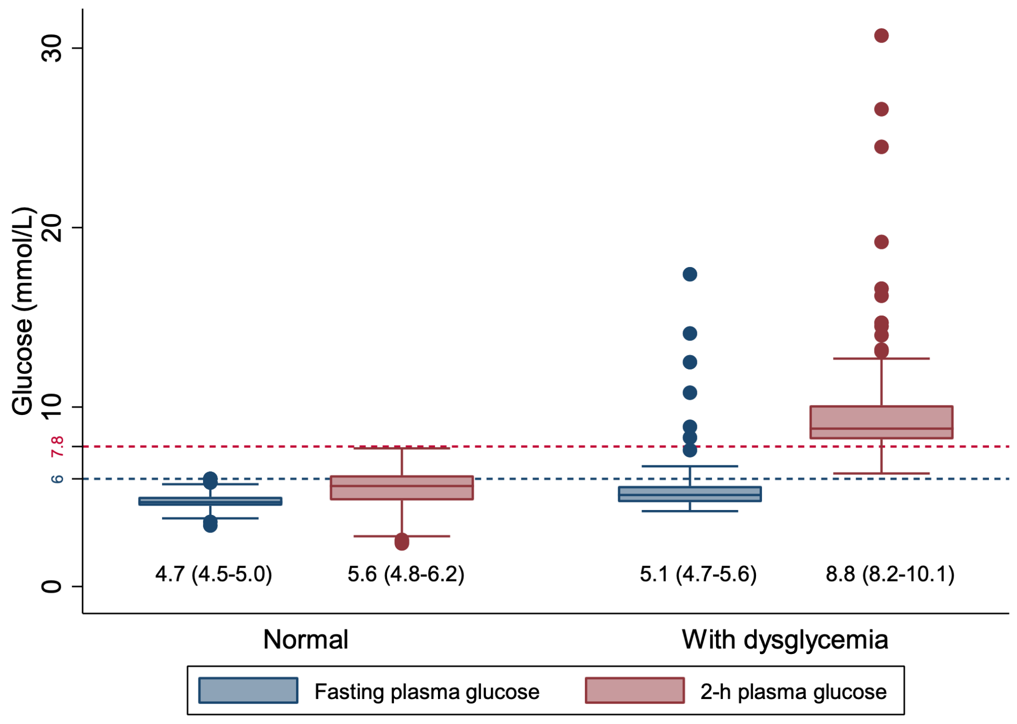

Figure S2: Boxplot distribution of fasting and 2h-glucose between women without (n=865) and with dysglycemia (n=106). Numbers below each boxplot represent median (interquartile range) values for fasting and 2-h plasma glucose (mmol/L).
Blue dashed line: fasting glucose cut-point 6.0 mmol/L; red dashed line: 2-h plasma glucose cut-point 7.8 mmol/L.

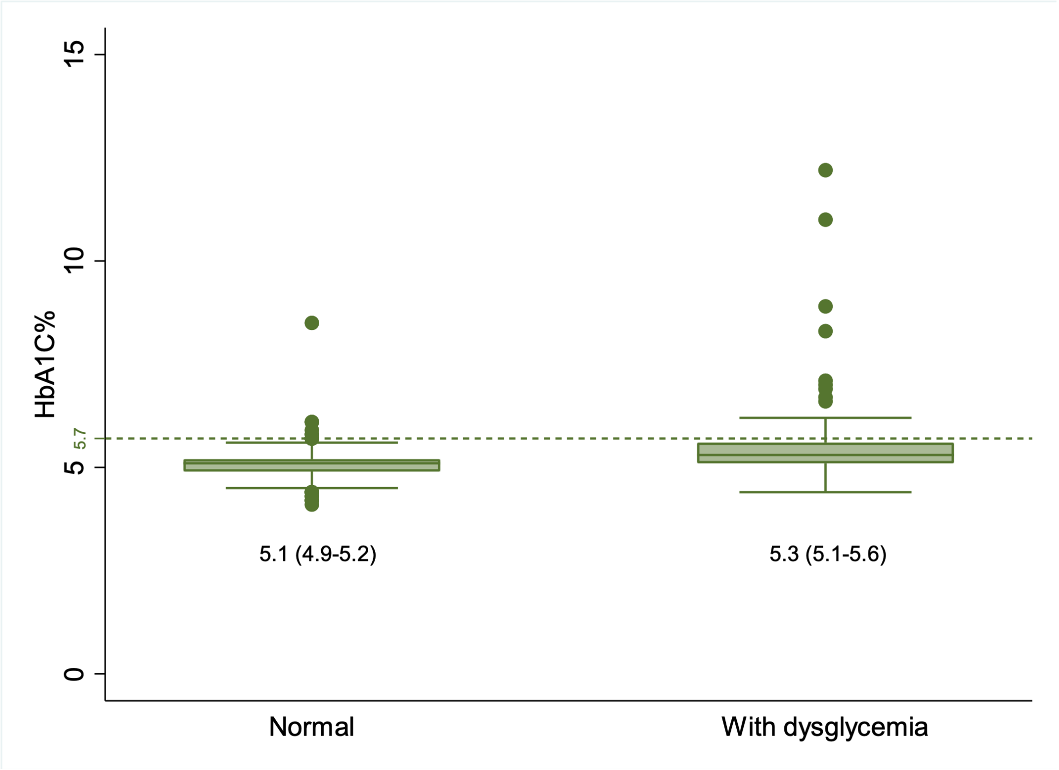

Figure S3: Boxplot distribution of HbA1c between women without (n=841) and with dysglycemia (n=105) women. Numbers below each boxplot represent median (interquartile range) values for HbA1c (%).

Table S1: Study inclusion and exclusion criteria

**Inclusion criteria**
Women aged 18-45 years
Intention to reside in Singapore for the next 5 years
Chinese, Malay, Indians, or a combination of these
Planning to conceive within 1 year
Able to provide written, informed consent
**Exclusion criteria**
Have been actively trying to conceive for more than 18 months
Currently pregnant<br>Type 1 or Type 2 diabetes
On fertility medication (e.g. hormones injection, IVF treatments) other than *Clomid / Letrozole (Femara)* in the past 1 month
Oral or implanted contraception, or with an IUCD in situ in the past 1 month
On systemic steroids in the past 1 month
On anticonvulsants in the past 1 month
On HIV or Hepatitis B or C treatment medication in the past 1 month
Eventual multiple pregnancies

Table S2: Inter-observer technical error of measurement (TE) and coefficient of variation (CV) of anthropometry

	**TE**	**CV (%)**
**Weight**	0.06 kg	0.13
**Height**	0.50 cm	0.26
**Waist circumference**	1.36 cm	2.20
**Hip circumference**	1.56 cm	2.39
**Triceps skinfolds**	0.49 mm	4.26
**Biceps skinfolds**	0.52 mm	13.29
**Subscapular skinfolds**	1.27 mm	11.80
**Suprailiac skinfolds**	1.63 mm	29.91

Table S3: Comparison of characteristics between included and excluded participants†

	Included n=971	Excluded n=62	p-value
**Demographics**			
Age (year)	30.8 ± 3.7	31.2 ± 4.7	0.46
**Ethnicity**			0.14
Chinese	698 (71.9)	42 (80.8)	
Malay	153 (15.8)	2 (3.8)	
Indian	87 (8.9)	6 (11.5)	
Mixed	33 (3.4)	2 (3.9)	
**Educational level**			0.01
No/Primary/Secondary	114 (11.7)	11 (25.0)	
Post-secondary	243 (25.0)	14 (31.8)	
Tertiary	614 (63.2)	19 (43.2)	
**Smoking history**			
Never	868 (89.4)	32 (88.8)	0.94
Previous smoker	60 (6.2)	2 (5.6)	
Current smoker	43 (4.4)	2 (5.6)	
**Shiftwork**‡			0.72
No/Unemployed	834 (85.9)	31 (83.8)	
Yes	137 (14.1)	6 (16.2)	
**Clinical characteristics**			
**Menstrual cycle regularity**			0.17
Regular	629 (64.8)	24 (54.5)	
Irregular	342 (35.2)	20 (45.5)	
**Parity**			0.66
Nulliparous	627 (64.6)	28 (80.0)	
Primiparous	262 (27.0)	5 (14.3)	
Multiparous	82 (8.4)	2 (5.7)	
**Previous history of GDM**§			0.36
No	466 (47.8)	14 (40.0)	
Yes	24 (2.5)	0 (0)	

**Family history of diabetes**			0.10
No	681 (70.1)	15 (55.6)	
Yes	290 (29.9)	12 (44.4)	
**Glucose test**			
HbA1c	5.1 ± 0.5	5.2 ± 0.5	0.29
FPG	4.8 ± 0.7	4.9 ± 1.0	0.47
2hPG	6.0 ± 2.1	6.8 ± 3.4	0.10

† Values are means ± SD or *n* (%).
‡ Only for subjects who were employed.
§ Excluding nulliparous women
Sample sizes differ due to missing data.
P-values obtained by independent t-test (continuous) or chi-square analysis/ Fisher's exact test (categorical).

Table S4: Anthropometric measures of all women by glycemic status

	All (%)† (n=971)	Normal Glucose Tolerance (n=865)	Dysglycemia (n=106)	p-value
Anthropometric measures§				
Weight (kg)	971 (100.0)	59.9 ± 13.1	67.2 ± 16.1	<0.01
Height (m)	971 (100.0)	1.60 ± 0.55	1.59 ± 0.57	<0.01
Waist circumference (cm)	968 (99.7)	82.5 ± 11.4	89.1 ± 12.8	<0.01
Hip circumference (cm)	968 (99.7)	96.4 ± 9.8	100.6 ± 11.5	<0.01
Triceps skinfolds (mm)	969 (99.8)	22.8 ± 7.5	27.2 ± 7.3	<0.01
Biceps skinfolds (mm)	969 (99.8)	11.1 ± 5.6	15.5 ± 7.5	<0.01
Subscapular skinfolds (mm)	959 (98.8)	20.1 ± 7.8	26.9 ± 9.0	<0.01
Supra-iliac skinfolds (mm)	968 (99.7)	18.0 ± 7.4	22.5 ± 7.7	<0.01
BMI (kg/m^2)	971 (100.0)	23.4 ± 5.0	26.7 ± 6.2	<0.01
Waist-to-hip ratio	967 (99.6)	0.85 ± 0.06	0.89 ± 0.06	<0.01
Waist-to-height ratio	968 (99.7)	0.52 ± 0.07	0.56 ± 0.08	<0.01
ABSI¶	968 (99.7)	0.0802 ± 0.0053	0.0799 ± 0.0046	0.58
Peripheral (triceps + biceps) skinfolds (mm)	969 (99.8)	34.0 ± 11.7	42.7 ± 13.8	<0.01
Truncal (subscapular + suprailiac) skinfolds (mm)	958 (98.7)	38.0 ± 14.0	49.3 ± 15.5	<0.01
Total skinfolds (mm)	958 (98.7)	71.8 ± 24.3	91.7 ± 27.6	<0.01
Subscapular-to-triceps ratio	959 (98.8)	0.89 ± 0.23	0.99 ± 0.22	<0.01

† Total sample size (n) is not always 971 due to the missing values
§ Mean ± SD
¶ Rounded to four decimal places due to small values
ABSI, A Body Shape Index; BMI, body mass index

Table S5: Sensitivity analysis of categorical anthropometry and HbA1c – AUROC, sensitivity and specificity in detecting dysglycemia with adjustment for four commonly considered risk factors (age, ethnicity, previous GDM history, and family history of diabetes)

			With history		
**Model**	**Variables**	**n**	**AUROC[†] (95% CI)**	**Sensitivity[‡] (%)**	**Specificity[‡] (%)**
1	History[§]	971	0.59 (0.53-0.64)[y]	47.2	65.6
2	HbA1c ≥5.7%	946	0.66 (0.60-0.72)[ax]	29.5	96.3
3	BMI ≥23kg/m^2	971	0.68 (0.63-0.73)[A]	73.6	57.1
4	WHtR ≥90th percentile	968	0.61 (0.55-0.67)[y]	31.1	81.1
5	Total skinfolds ≥90th percentile	958	0.65 (0.59-0.71)[a]	33.3	86.1
6	BMI ≥23kg/m^2 + WHtR ≥90th percentile + Total skinfolds ≥90th percentile	957	0.71 (0.66-0.76)[Ay]	72.4	58.8
7	BMI ≥23kg/m^2 + HbA1c ≥5.7%	946	0.73 (0.67-0.78)[Axy]	63.8	65.2
8	BMI ≥25kg/m^2 + HbA1c ≥5.7%	946	0.74 (0.69-0.80)[Axy]	63.8	74.3
9	BMI ≥23kg/m^2 + WHtR ≥90th percentile + Total skinfolds ≥90th percentile + HbA1c ≥5.7%	932	0.75 (0.69-0.80)[Axy]	64.4	71.5

[†] AUROC values were based on cases containing only complete data on all variables (n=932).
[‡] Sensitivity and specificity estimates were based on cases with available variables used to create each model.
[§] Age (>30 years), ethnicity, GDM history, and family history of diabetes
[a] $p<0.05$; [A] $p<0.001$ vs Model 1 (history)
[x] $p<0.05$ vs Model 2 with history
[y] $p<0.05$ vs Model 3 with history
AUROC, area under receiver-operating characteristic; BMI, body mass index; HbA1c, glycated haemoglobin; WHtR, waist-to-height ratio

Table S6: Clinical utility of different strategies in detecting dysglycemia preconception among 922 women without previous history of GDM who provided both BMI and HbA1C measures (99 out of 922 women with a HbA1c measure had dysglycemia)

		Positive predictive value (%)	Negative predictive value (%)	Number to proceed to OGTT (% of all screened)	Number of dysglycemia identified (% of all dysglycemia)	Number of dysglycemia cases missed (% of all dysglycemia)
1.	HbA1c≥5.7%	65.8	91.6	38 (4.1%)	25 (25.3%)	74 (74.7%)
2.	BMI≥23kg/m^2 +HbA1c≥5.7%	17.2	94.6	418 (46.0%)	72 (72.7%)	27 (27.3%)
3.	BMI ≥23kg/m^2 + WHtR ≥90th percentile + Total skinfolds ≥90th percentile + HbA1c ≥5.7%†	17.9	94.3	380 (41.8%)	68 (69.4%)	30 (30.6%)
4.	BMI≥25kg/m^2 +HbA1c≥5.7%	22.9	94.2	266 (28.9%)	61 (61.6%)	38 (38.4%)
5.	BMI ≥23kg/m^2 + HbA1c ≥5.7% + History‡	21.1	93.7	275 (29.8%)	58 (58.6%)	41 (41.4%)

† n=909 provided all measurements (98 out of 909 women with dysglycemia).
‡ History variables included: Age (>30 years), ethnicity, educational level, smoking history, shift work, menstrual cycle regularity, parity, and family history of diabetes.

Table S7: Different thresholds of anthropometry and HbA1c for screening dysglycemia

	Without history			With history†		
	**AUROC (95% CI)**	**Sensitivity (%)**	**Specificity (%)**	**AUROC (95% CI)**	**Sensitivity (%)**	**Specificity (%)**
**BMI (kg/m²)**						
**0.5 unit increment**						
≥27.5	0.624 (0.58-0.67)	40.6	84.3	0.702 (0.65-0.76)	58.5	70.6
≥27	0.631 (0.58-0.68)	43.4	82.8	0.706 (0.65-0.76)	61.3	69.5
≥26.5	0.646 (0.60-0.70)	48.1	81.2	0.715 (0.66-0.77)	65.1	70.9
≥26	0.653 (0.60-0.70)	51.9	78.6	0.719 (0.67-0.77)	67.9	69.7
≥25.5	0.667 (0.62-0.72)	56.6	76.9	0.721 (0.67-0.78)	64.2	73.1
≥25	0.672 (0.62-0.72)	59.4	75.0	0.727 (0.67-0.78)	63.2	72.4
≥24.5	0.656 (0.61-0.70)	59.4	71.7	0.719 (0.67-0.77)	65.1	68.7
≥24	0.667 (0.62-0.71)	66.0	67.3	0.719 (0.67-0.77)	67.9	66.0
≥23.5	0.668 (0.62-0.71)	69.8	63.8	0.724 (0.67-0.78)	71.7	63.4
≥23	0.648 (0.60-0.69)	71.7	57.9	0.707 (0.65-0.76)	67.9	68.7
**0.1 unit increment**						
≥24.0	0.667 (0.62-0.71)	66.0	67.3	0.719 (0.67-0.77)	67.9	66.0
≥23.9	0.670 (0.62-0.72)	67.0	66.9	0.723 (0.67-0.78)	68.9	65.6
≥23.8	0.671 (0.62-0.72)	67.9	66.2	0.726 (0.67-0.78)	69.8	64.9
≥23.7	0.673 (0.63-0.72)	68.9	65.8	0.729 (0.68-0.78)	70.8	64.5
≥23.6	0.670 (0.62-0.72)	68.9	65.2	0.728 (0.68-0.78)	70.8	64.3
≥23.5	0.668 (0.62-0.71)	69.8	63.8	0.724 (0.67-0.78)	71.7	63.4
≥23.4	0.665 (0.62-0.71)	70.8	62.3	0.722 (0.67-0.77)	72.6	62.4
≥23.3	0.664 (0.62-0.71)	71.7	61.2	0.721 (0.67-0.77)	72.6	61.9
≥23.2	0.657 (0.61-0.7)	71.7	59.8	0.713 (0.66-0.77)	69.8	62.9
≥23.1	0.652 (0.61-0.7)	71.7	58.7	0.709 (0.65-0.76)	67.9	66.7
≥23.0	0.648 (0.6-0.69)	71.7	57.9	0.707 (0.65-0.76)	67.9	68.7
**WHtR percentile**						
≥99	0.525 (0.50-0.55)	1.9	99.2	0.653 (0.60-0.71)	55.7	66.8
≥95	0.566 (0.53-0.61)	9.4	95.5	0.654 (0.60-0.71)	53.8	66.9
**Total Skinfolds percentile**						
≥99	0.563 (0.53-0.60)	2.9	99.3	0.656 (0.60-0.71)	55.2	66.6
≥95	0.511 (0.49-0.53)	16.2	96.4	0.674 (0.62-0.73)	49.5	74.0
**HbA1c (%)**						
≥5.6	0.632 (0.59-0.68)	29.5	96.9	0.720 (0.66-0.78)	53.3	78.8
≥5.5	0.651 (0.60-0.70)	36.2	94.1	0.728 (0.67-0.79)	56.2	78.6
≥5.4	0.684 (0.63-0.73)	48.6	88.2	0.753 (0.70-0.81)	59.1	82.1
≥5.3	0.701 (0.65-0.75)	61.0	79.3	0.763 (0.71-0.81)	68.6	75.9
≥5.2	0.695 (0.65-0.74)	74.3	64.7	0.755 (0.71-0.80)	77.1	63.9
≥5.1	0.656 (0.62-0.69)	84.8	46.5	0.728 (0.68-0.77)	73.3	61.9

≥5.0	0.581 (0.55-0.61)	89.5	26.8	0.687 (0.64-0.74)	66.7	59.3

†Age (>30 years), ethnicity, educational level, smoking history, shift work, menstrual cycle regularity, parity, GDM history, and family history of diabetes. AUROC, area under receiver-operating characteristic; BMI, body mass index; HbA1c, glycated hemoglobin; WHtR, waist-to-height ratio

Table S8: Sensitivity analyses performed using different BMI (≥23.7 and ≥27.5 kg/m²) and HbA1c cut-offs (≥5.3 and ≥6.0%) in detecting dysglycemia

		Without history			With history		
**Model**		**AUROC (95% CI)**	**Sensitivity (%)**	**Specificity (%)**	**AUROC (95% CI)**	**Sensitivity (%)**	**Specificity (%)**
1	History†	-	-	-	0.65 (0.60-0.71)	55.7	67.3
**BMI ≥23.7 kg/m²**							
2	BMI ≥23.7 kg/m²	0.68 (0.63-0.72)[z]	68.9	65.8	0.73 (0.68-0.78)[a]	70.8	64.5
**BMI ≥27.5 kg/m²**							
3	BMI ≥27.5 kg/m²	0.63 (0.58-0.68)[z]	40.6	84.3	0.70 (0.65-0.76)[a]	58.5	70.6
4	BMI ≥27.5 kg/m² + WHtR ≥90th percentile + Total skinfolds ≥90th percentile	0.64 (0.59-0.69)[z]	41.9	83.9	0.71 (0.66-0.76)[a]	58.1	72.1
5	BMI ≥27.5 kg/m²+ HbA1c ≥5.7%	0.67 (0.62-0.73)[bz]	47.6	83.9	0.74 (0.68-0.79)[aw]	55.2	77.1
6	BMI ≥27.5 kg/m² + Total skinfolds ≥90th + WHtR ≥90th percentile + HbA1c ≥5.7%	0.68 (0.63-0.74)[bz]	46.2	86.1	0.73 (0.68-0.79)[aw]	53.9	79.8
**HbA1c ≥5.3%**							
7	HbA1c ≥5.3%	0.70 (0.65-0.75)[z]	61.0	79.3	0.76 (0.71-0.81)[a]	68.6	76.0
8	BMI ≥23 kg/m² + HbA1c ≥5.3%	0.74 (0.69-0.79)[acz]	61.0	79.3	0.77 (0.73-0.82)[a]	67.6	75.7
9	BMI ≥23.7 kg/m² + HbA1c ≥5.3%	0.75 (0.69-0.8)[acz]	61.0	79.3	0.78 (0.73-0.83)[a]	66.7	74.9
10	BMI ≥23.7 kg/m² + WHtR ≥90th percentile + Total skinfolds ≥90th percentile + HbA1c ≥5.3%	0.75 (0.70-0.81)[acz]	65.4	75.7	0.78 (0.73-0.83)[a]	69.2	75.7
**HbA1c ≥6.0%**							
11	HbA1c ≥6.0%	0.58 (0.54-0.62)[az]	17.1	99.6	0.69 (0.64-0.75)[a]	52.4	75.3
12	BMI ≥23 kg/m² + HbA1c ≥6.0%	0.69 (0.63-0.74)[dz]	72.4	57.9	0.74 (0.69-0.80)[ay]	61	73.4
13	BMI ≥23 kg/m² + WHtR ≥90th percentile + Total skinfolds ≥90th percentile + HbA1c ≥6.0%	0.70 (0.64-0.75)[dz]	72.1	58.5	0.74 (0.69-0.80)[ay]	60.6	73

† Age (>30 years), ethnicity, educational level, smoking history, shift work, menstrual cycle regularity, parity, GDM history, and family history of diabetes
[a] p<0.05 vs Model 1 (history)
[b] p<0.05 vs Model 3 without history (across Model 3 – 6)
[c] p<0.05 vs Model 7 without history (across Model 7 –10)
[d] p<0.05 vs Model 11 without history (across Model 11 –13)
[w] p<0.05 vs Model 3 with history (across Model 3 – 6)
[x] p<0.05 vs Model 7 with history (across Model 7 –10)
[y] p<0.05 vs Model 11 with clinical history (across Model 11 –13)
[z] p<0.05 vs with history
AUROC, area under receiver-operating characteristic; BMI, body mass index; HbA1c, glycated hemoglobin; WHtR, waist-to-height ratio
